# Supplementary material for: Sourdough Bread with Different Fermentation Times: A Randomized Clinical Trial in Subjects with Metabolic Syndrome
Source: Nutrients. 2024 Jul 23;16(15):2380. doi: 10.3390/nu16152380 (PMC11314010; doi:10.3390/nu16152380)
Supplement: Supplementary file 1 [file nutrients-16-02380-s001.zip › nutrients-3084151-supplementary.pdf]

Sourdough Bread with Different Fermentation Times: A Randomized Clinical Trial in Subjects with Metabolic Syndrome

Supplementary material

Supplementary Table S1: Nutritional composition of EBLong and EBShort breads. ………………………..2
Supplementary Table S2: Intra and inter-assay coefficient of variation for the inflammatory and satiety-related biomarkers. ………………………..2
Supplementary Table S3: Difference in dietetic assessment of food records in EBShort and EBLong groups. ………………………..3
Supplementary Figure S1: Alpha and beta diversity of EBShort and EBLong intervention. ………………………..4
Supplementary Table S4: Differential abundance at phylum taxonomic level. ………………………..5

Supplementary Table S1: Nutritional composition of EBLong and EBShort breads.

	**EBLong**	**EBShort**
Energy (kcal/100g)	245.33	257.67
Fat (g/100g)	0.98	0.75
Saturated Fat (g/100g)	0.40	0.13
Monounsaturated Fat (g/100g)	0.13	0.17
Polyunsaturated Fat (g/100g)	0.40	1.60
Carbohydrate (g/100g)	49.43	51.33
Sugar (g/100g)	2.40	1.10
Fiber (g/100g)	3.13	2.60
Proteins (g/100g)	8.12	10.12
Sodium (g/100g)	0.74	0.50

EBLong, Elias Boulanger® long fermentation bread; EBShort, Elias Boulanger® short fermentation bread.

Supplementary Table S2: Intra and inter-assay coefficient of variation for the inflammatory and satiety-related biomarkers.

**Biochemical determinations**	**Intra- assay CV (%)**	**Inter- assay CV (%)**
Inflammatory biomarkers		
IL6, pg/mL	4.78	6.69
IL8, pg/mL	8.38	4.86
TNF-α, pg/mL	3.75	-
PAI-1, pg/mL	0.78	12.55
sICAM, pg/mL	11.3	6.31
LBP, ng/mL	2.21	2.59
Satiety-related hormones		
Insulin, pg/mL	5.30	1.09
Glucagon, pg/mL	6.61	17.88
GLP-1, pg/mL	16.82	1.69
Visfatin, pg/mL	22.47	1.53
Resistin, pg/mL	2.98	9.10
C-peptide, pg/mL	7.27	7.19
Ghrelin, pg/mL	0.99	10.88
Leptin, pg/mL	6.46	0.07

CV, coefficient of variation.

Supplementary Table S3: Difference in dietetic assessment of food records in EBLong and EBShort groups.

	EBLong			EBShort			Intergroup comparison (EBLong vs EBShort)			
	**Baseline**	**Follow-up**	***p* value**	**Baseline**	**Follow-up**	***p* value**	**Non-adjusted (diff. [95% CI])**	***p* value**	**Adjusted (diff. [95% CI])**	***p* value**
n	12	9		17	16					
Energy, kcal/day	1570 (291)	1610 (287)	0.422	1550 (387)	1590 (436)	0.696	-26 [-345; 293]	0.874	145 [-230; 520]	0.460
Fats, g/day	72.7 (19.1)	67.5 (18.6)	0.762	68.1 (20.3)	67.1 (19.5)	0.829	-0.4 [-16.1; 15.3]	0.960	18.2 [-3.76; 40.2]	0.125
SFA, g/day	19.1 (7.54)	17.1 (8.18)	0.389	17 (6.13)	17.7 (5.62)	0.609	0.55 [-4.86; 5.96]	0.843	4.5 [-1; 10]	0.129
Cholesterol, g/day	221 (58.9)	236 (81.9)	0.792	271 (101)	260 (70.3)	0.670	24.7 [-36.2; 85.5]	0.435	7.9 [-56; 71.8]	0.812
Carbohydrates, g/day	143 (36.2)	164 (36.5)	0.120	144 (38.2)	146 (38.8)	0.883	-17.1 [-48.1; 13.9]	0.292	-23.4 [-57.3; 10.4]	0.195
Sugars, g/day	61.7 (19.8)	62.9 (12.9)	0.541	58.5 (15.8)	59.4 (19.3)	0.658	-3.44 [-17.6; 10.7]	0.638	2.84 [-18.3; 23.9]	0.795
Fiber, g/day	17.8 (5.73)	18.2 (4.69)	0.905	18.7 (6.92)	17.6 (4.4)	0.448	0.56 [-3.18; 4.31]	0.770	1.19 [-2.62; 4.99]	0.548
Alcohol, g/day	1.95 (2.94)	2.1 (4.06)	0.664	4.31 (6.89)	4.9 (10.6)	0.539	2.8 [-4.48; 10.1]	0.458	2.58 [-5.49; 10.7]	0.540
Proteins, g/day	77.7 (13.6)	81.3 (18)	0.330	79.5 (19.3)	84 (28.5)	0.517	2.75 [-17.9; 23.4]	0.797	11.3 [-13.7; 36.2]	0.390

Baseline and follow-up values are presented as mean (SD). EBLong, Elias Boulanger® long fermentation bread; EBShort, Elias Boulanger® short fermentation bread; SFA: Saturated fatty acids. Baseline and follow-up values are presented as mean and standard deviation (SD). Intergroup comparisons in follow-up values relative to control group were estimated by multivariable linear regression adjusted for age, sex, BMI, MedDiet adherence (14pt) and baseline values.

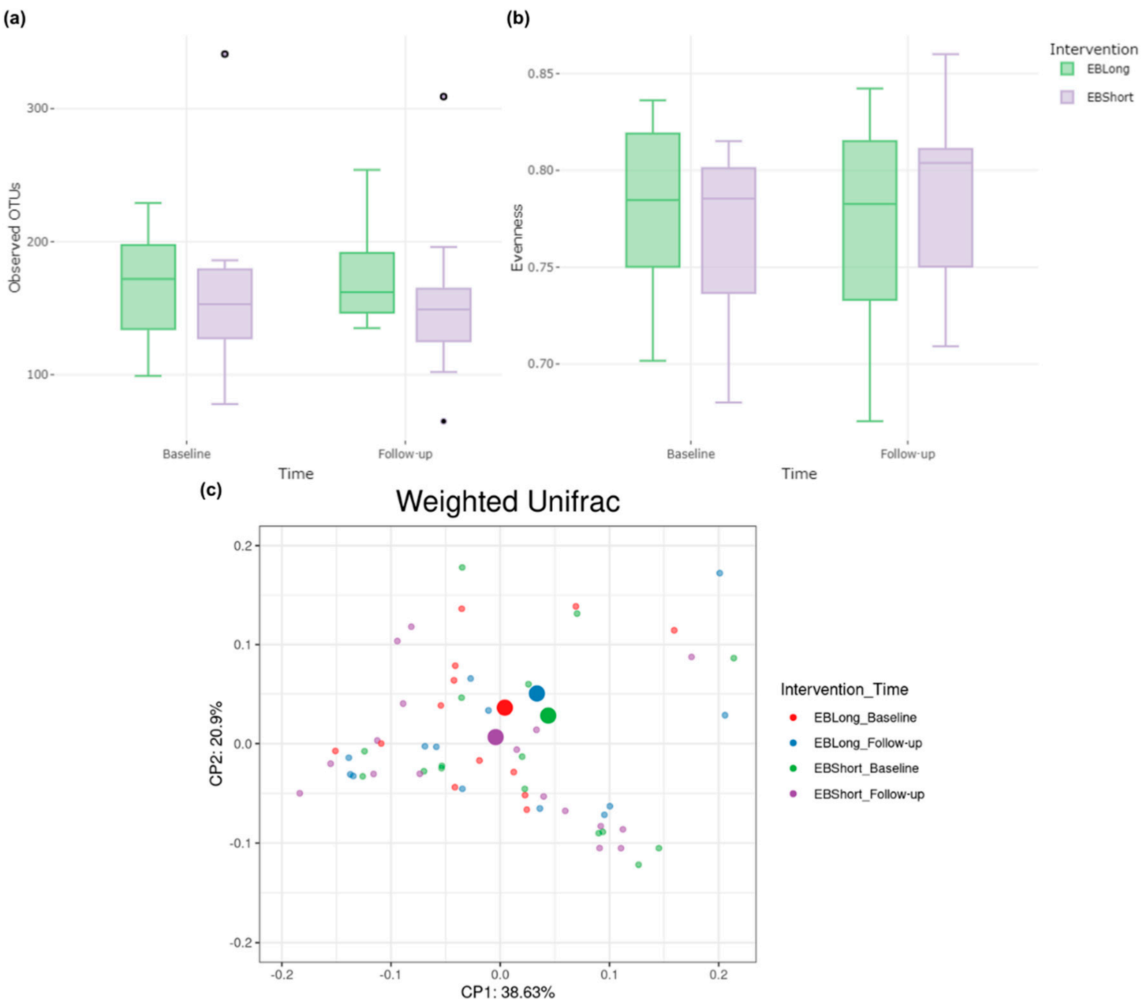

Supplementary Figure S1: Alpha and beta diversity of EBLong and EBShort intervention. (a) Diversity richness as the number of observed OTUs at baseline and at follow up in each intervention. (b) Pielou's evenness index in each intervention groups at baseline and at follow up. (c) Weighted UniFrac distances according to interventions and follow up.

Supplementary Table S4: Differential abundance at phylum taxonomic level.

	EBLong				EBShort				EBLong vs EBShort							
	Baseline vs Follow-up				Baseline vs Follow-up				Baseline				Follow-up			
**Phylum**	**Est**	**SE**	**T ratio**	**p value**	**Est**	**SE**	**T ratio**	**p value**	**Est**	**SE**	**T ratio**	**p value**	**Est**	**SE**	**T ratio**	**p value**
Euryarchaeota	0.413	0.230	1.793	0.084	0.350	0.196	1.781	0.086	-0.140	0.810	-0.172	0.864	-0.203	0.820	-0.247	0.807
Actinobacteriota	-0.195	0.208	-0.938	0.356	0.174	0.182	0.952	0.349	0.269	0.311	0.867	0.393	0.639	0.311	2.056	0.050
Bacteroidota	0.101	0.175	0.576	0.570	-0.137	0.153	-0.894	0.379	0.200	0.177	1.131	0.268	-0.037	0.177	-0.209	0.836
Cyanobacteria	-0.998	0.330	-3.020	0.005	-1.016	0.341	-2.979	0.006	-0.027	1.098	-0.024	0.981	-0.046	1.050	-0.043	0.966
Desulfobacterota	-0.185	0.251	-0.736	0.468	0.158	0.254	0.622	0.539	0.218	0.484	0.451	0.655	0.561	0.484	1.159	0.257
Firmicutes	-0.030	0.077	-0.389	0.700	0.075	0.067	1.121	0.272	-0.097	0.085	-1.140	0.264	0.008	0.085	0.095	0.925
Patescibacteria	0.051	0.480	0.106	0.916	-0.206	0.300	-0.686	0.499	-0.559	0.601	-0.930	0.361	-0.815	0.598	-1.364	0.184
Proteobacteria	0.284	0.282	1.007	0.323	-0.101	0.247	-0.409	0.685	0.728	0.477	1.526	0.139	0.343	0.477	0.718	0.479
Synergistota	3.911	0.905	4.319	<0.001	-0.036	0.246	-0.145	0.886	0.395	1.073	0.368	0.716	-3.551	1.390	-2.556	0.017
Verrucomicrobiota	0.452	0.274	1.647	0.111	-0.267	0.184	-1.450	0.158	0.021	0.734	0.028	0.978	-0.698	0.741	-0.942	0.355

EBLong, Elias Boulanger® long fermentation bread; EBShort, Elias Boulanger® short fermentation bread; Est: Estimate, SE: Standard error. P-values were adjusted using false discovery rate (FDR).
